# A toolbox for real-time subject-independent and subject-dependent classification of brain states from fMRI signals

**DOI:** 10.3389/fnins.2013.00170

**Published:** 2013-10-17

**Authors:** Mohit Rana, Nalin Gupta, Josue L. Dalboni Da Rocha, Sangkyun Lee, Ranganatha Sitaram

**Affiliations:** ^1^Institute of Medical Psychology and Behavioral Neurobiology, University of TübingenTübingen, Germany; ^2^Graduate School of Neural & Behavioural Sciences International Max Planck Research School, University of TübingenTübingen, Germany; ^3^Department of Biomedical Engineering, University of FloridaGainesville, FL, USA; ^4^Electronics and Electrical Communication, Indian Institute of Technology KharagpurKharagpur, India; ^5^Max Planck Institute for Biological CyberneticsTübingen, Germany; ^6^Sree Chitra Tirunal Institute for Medical Sciences & TechnologyTrivananthapuram, India

**Keywords:** fMRI BOLD, SVM, neurofeedback, classification, real-time fMRI, pattern recognition, multivariate analysis

## Abstract

There is a recent increase in the use of multivariate analysis and pattern classification in prediction and real-time feedback of brain states from functional imaging signals and mapping of spatio-temporal patterns of brain activity. Here we present MANAS, a generalized software toolbox for performing online and offline classification of fMRI signals. MANAS has been developed using MATLAB, LIBSVM, and SVMlight packages to achieve a cross-platform environment. MANAS is targeted for neuroscience investigations and brain rehabilitation applications, based on neurofeedback and brain-computer interface (BCI) paradigms. MANAS provides two different approaches for real-time classification: subject dependent and subject independent classification. In this article, we present the methodology of real-time subject dependent and subject independent pattern classification of fMRI signals; the MANAS software architecture and subsystems; and finally demonstrate the use of the system with experimental results.

## Introduction

### Pattern classification and rationale behind choosing SVM

Prediction of brain states from functional imaging constitutes a major scope of neuroscience with applications ranging from neuro-rehabilitation, brain-computer interfacing, neuro-feedback in understanding brain function during cognition, perception, and detection of deception (Haynes and Rees, 2006). Recent studies in pattern classification of functional magnetic resonance imaging (fMRI) signals have suggested the need for multivariate pattern classification. A major argument in favor of multivariate pattern classification is that perceptual, cognitive, and emotional processes generally recruit a distributed network of brain regions rather than a single location. However, univariate statistical parametric mapping, traditionally used in functional imaging, compares activation levels at each voxel between a task state and a baseline state, or another task state, in order to determine whether the voxel is involved in a particular task or not. This approach does not take into account the interaction between multiple brain regions in a task. On the other hand, multivariate methods consider activation levels at multiple distributed voxels, to identify regions that are involved in distinct tasks. Furthermore, multivariate methods allow for trial-by-trial decoding of the task condition the brain is involved in, enabling a number of novel approaches to real-time brain state prediction and feedback.

A number of methodological studies have applied pattern classification to analyze fMRI signals offline with a view to map the pattern of a variety of tasks involved in cognitive, perceptual, motor, and emotion processes (LaConte et al., 2003, 2005; Shaw et al., 2003; Strother et al., 2004; Martinez-Ramon et al., 2006). LaConte et al. (2003) examined the classification of block-design fMRI data using linear discriminant analysis (LDA) and Support Vector Machines (SVM) in contrast to canonical variates analysis (CVA). Mourao-Miranda et al. (2006) compared SVM and the Fisher linear discriminant (FLD) classifier and demonstrated that SVM outperforms FLD in prediction accuracy as well as in robustness of the spatial maps obtained. SVM has also been shown to have certain advantages in the classification of fMRI signals in comparison to other methods such as LDA (LaConte et al., 2005) and multilayer neural networks. SVM is less sensitive to preprocessing (LaConte et al., 2005), is better capable of handling large data size (Haynes and Rees, 2006), and most importantly produces unique optimal solutions (Collobert et al., 2006). Although SVM model training is computationally intensive, current availability of faster, yet cheaper processors compensate for this drawback (LaConte et al., 2007).

LaConte et al. (2007) reported the first real-time fMRI system with multivariate classification for decoding motor imagery and demonstrated the classifier's ability to decode other forms of cognitive and emotional states. Later, Sitaram et al. (2011) developed a robust method, based on effect mapping (Lee et al., 2010), for real-time prediction and feedback of multiple brain states, and demonstrated its power in online classification of emotional brain states and control of brain activity by feedback training.

Massive On-line Analysis (MOA) (Bifet et al., 2010) and Shogun (Sonnenburg et al., 2010) are two toolboxes available for performing real-time classification of fMRI data. These toolboxes provide a wide range of dimensionality reduction algorithms and provide feedback to the subject based on virtual reality. These toolboxes have a limitation to integrate with Matlab in order to use classification data for providing feedback to subjects that can increase the flexibility in designing the experiment. Motivated by the emerging interest and rapid developments in the multivariate classification of brain states for varied applications, and limitation of these toolboxes, we have developed an integrated toolbox for offline and online classification and feedback of multiple brain states from fMRI signals. Furthermore, we have also introduced a novel approach, hitherto unreported, to classifying brain states of new participants whose brain signals have never been used to train the SVM classifier, in a method called Subject-independent Classification (SIC).

### Why is subject-independent classification necessary?

A major limitation of the current methods in the field of real-time fMRI is the lack of a suitable implementation of real-time SIC of brain states. Existing methods are suited for specific participants due to the fact that SVM classifiers are pre-trained on subject-specific data before being applied to the same participants for neuro-feedback or BCI training. However, such an approach has three major disadvantages:

1. Collecting data for classifier training for each subject is time consuming and requires the subject's involvement.
2. In neuro-rehabilitation applications with patients, it is improper to build a classifier on patient's abnormal brain activity, as feedback-based reinforcement of such abnormal brain states may further deteriorate the condition of the patient. What might be more appropriate would be to administer feedback training in patients from a classifier constructed from healthy subject's fMRI signals while performing the task that is being trained.
3. Subject data for training might not always be available, as in case of lie-detection and other security applications.

Thus, it becomes essential to develop a subject-independent classifier that could be applied to the new subjects' data without prior classifier training, and in addition, could be adapted to the idiosyncrasies of every individual's brain size, shape, and activation pattern.

Such a subject-independent classifier could then be used in clinical rehabilitation where patients with brain abnormalities pertaining to motor, cognitive or emotional processing could be retrained to achieve normal level of functioning by providing feedback from a real-time pattern classifier that is trained on healthy subjects. By repeated training and contingent reward from the classifier, patients could perhaps learn to mimic the brain activation of healthy individuals in order to ameliorate their problem.

### What are the other benefits of the toolbox?

An essential technical improvisation is required in realizing a subject-independent real-time classifier that will be to implement a mechanism of normalizing the functional images to a standard space [such as the Montreal Neurological Institute (MNI) standard space] in real-time so that inter-subject variations in brain size, shape, and activations can be ameliorated. To our knowledge, this is the first real-time classification toolbox that provides such functionality. In our previous studies, we demonstrated an online subject-dependent classifier for multi-class brain state classification and feedback (Sitaram et al., 2011). The present toolbox intends to integrate these different approaches and provide graphical user interfaces (GUI) for subject-dependent and subject-independent classification.

The design of the toolbox is such that it is not limited only to specific use-cases. Speaking at a broader level, the toolbox is meant to be used for any type of brain state multivariate classification, be it two-class or multi-class, online or offline, and subject-independent or dependent classification approaches.

In this paper first, we outline the design object for toolbox. Following this, we will discuss the reasons for the development of the various features of this toolbox, and the theory behind SVM classification by using effect mapping method (Lee et al., 2010) both for online and offline analysis. We will then outline the hypothesis and the result of the different data set, which are analyzed using this toolbox. We will conclude by describing the limitations and future directions of this toolbox.

## Software architecture

### Organization of the toolbox

As mentioned earlier, the main motivation for creating the toolbox was to provide a simple user interface to perform real-time multivariate analysis of fMRI signals for use in clinical rehabilitation as well as in neuroscience research. This may necessitate conducting analysis on the previously recorded data. Such a requirement has also been taken into consideration while designing the toolbox. The toolbox is aimed at achieving the following objectives:

1. To provide flexibility to perform both online and offline classification.
2. To be applicable for subject-independent analysis and subject-dependent analysis.
3. To act as a single toolbox for performing all steps including pre-processing, training, classification, and feedback.
4. To enable multi-class classification of fMRI signals.
5. To perform real-time pre-processing of fMRI images, such as realignment, co-registration, segmentation and normalization, for use in classification of brain states or brain mapping.

The toolbox works on the MATLAB 6.5 (The Mathworks, Natick, MA) software package, and uses code segments from Statistical Parametric Mapping (SPM2, University College London) for fMRI preprocessing and normalization, and LibSVM (http://www.csie.ntu.edu.tw/~cjlin/libsvm/) or SVMLight (Joachims et al., 1999) for SVM classification algorithms.

### Subsystems

The toolbox comprises of four sub-systems. These subsystems are an integral part of any experiment or use-case, be it online or offline, subject-independent or dependent, feedback or without feedback. However, the implementation of each of these sub-systems differs for each of the above use-cases, which will be described below.

#### Image acquisition

Because the toolbox is meant for both online and offline purposes, it needs to be generalized for different fMRI pulse sequences and scanner parameters. The fMRI images can be provided to the toolbox either directly from the scanner for online classification, or from a storage media for offline classification. The toolbox takes around 0.7–1.2 s to completely process a whole functional brain image, starting from image acquisition, pre-processing, and ending with classification. Given this computational time requirement for classification, the current version is best used for real-time classification and feedback when TR over 1 s.

The input files can either be raw images (either directly from the scanner or from a storage media) or preprocessed “swr” (smoothed, normalized, and realigned) images. Only raw scans need to be preprocessed.

#### Pre-processing

This is the first step toward analysis of the brain signals. It involves reading the DICOM files, converting them into Analyze format which is understood by the SPM2-based preprocessing scripts used in this toolbox, removing the noise in the acquired images, realigning them with each other, normalizing them (if required, as in the case of subject-independent analysis) to a standard space, and finally smoothing them. The images obtained after the pre-processing steps are then ready for analysis.

### Offline subject-dependent and subject-independent classification

**Figure 1:**
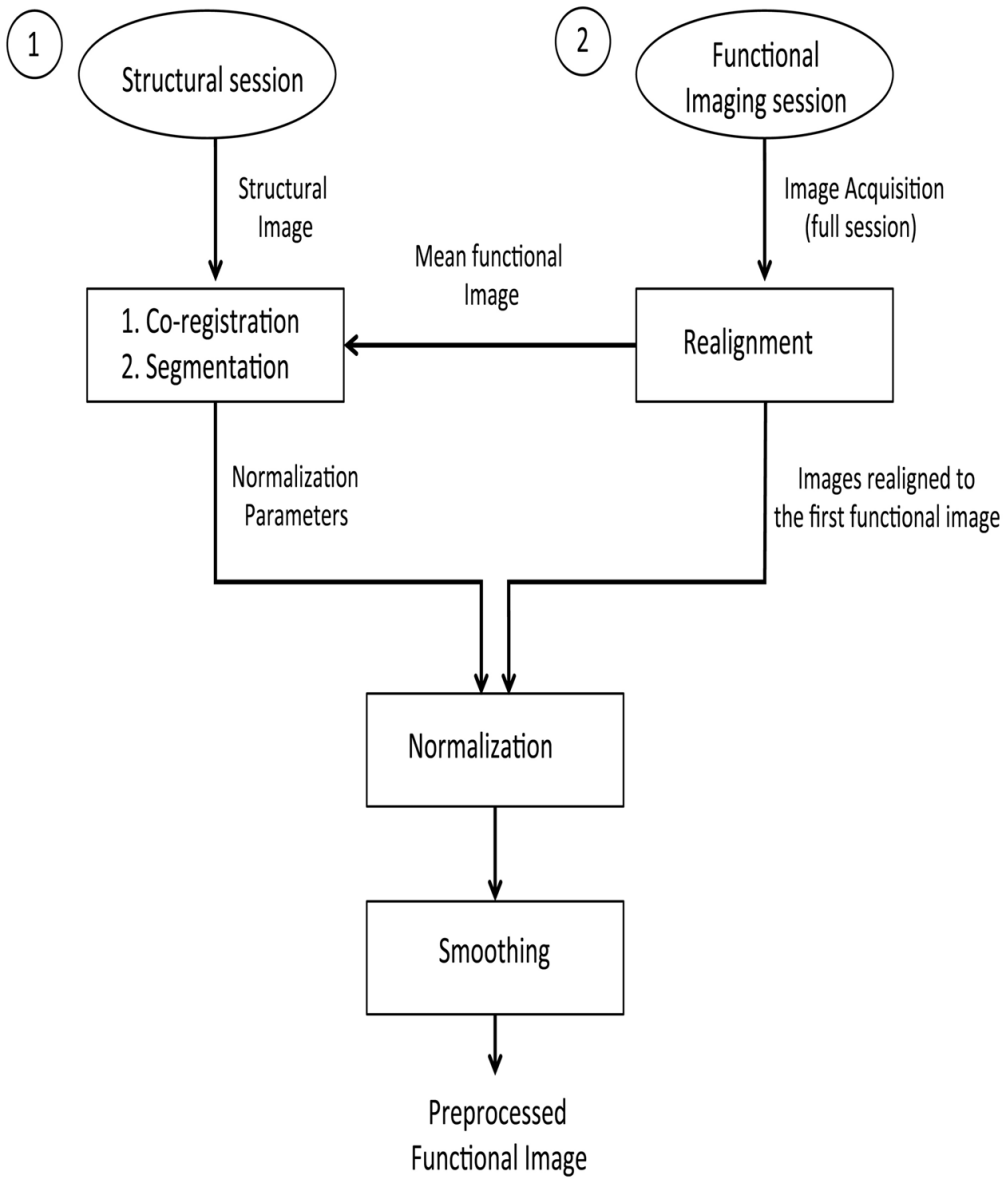
**Pre-processing steps on the structural and functional images for the offline subject-independent and dependent classifier**. First, re-alignment of the functional images is performed by considering the first functional image as the template. Next, co-registration is performed using the structural image and the mean functional image which is, created after the realignment of functional images. Following the segmentation of the structural image, normalization of the functional images is performed using the normalized parameters calculated in the previous step. Finally, functional images are smoothed using a Gaussian kernel.

The pre-processing steps involved in these analyses are presented in the form of a flowchart (Figure 1). First, the stored DICOM formatted (.dcm) images are converted to ANALYZE format resulting in two files in.img and.hdr format for each.dcm file. These two files store the actual data and the header information, respectively. To correct the head motion related artifacts, these raw fMRI images need to be realigned with respect to a representative scan (which, in most cases, is the first scan of the first session). The realignment subsystem discussed earlier is used for estimating the realignment parameters of the individual fMRI scan, which are stored in their individual.mat files. To estimate the transformation (normalization) parameters, a reference brain image needs to be co-registered with the template. However, functional images are not suitable for this purpose because of their low spatial resolution. High-resolution T1 weighted structural images are used for estimating the normalization parameters. The mean functional fMRI image, which is created after the realignment step, is then co-registered with the structural scan. During this co-registration process, the structural image is modified to best fit the mean (representative) fMRI image. It is easier to modify one structural image than modifying several functional images. The modified structural image is then segmented using the prior tissue probability maps. The spatial normalization parameters are estimated and stored in a.mat file.

Given that fMRI images are realigned to the mean functional image, which is in turn co-registered with a structural image whose normalization parameters are known, we can apply the matrix chain rule to normalize the fMRI images. This involves a multiplication of the fMRI realignment parameters and the normalization parameters found upon segmentation. The raw fMRI images are finally re-sliced using these parameters found upon the multiplication process. There are two advantages in adopting this method of combining the realignment parameters and normalization parameters into a single matrix: the re-slicing operation is executed only once. Had the normalization parameters been applied over the fMRI images (if they were previously re-sliced using the realignment parameters), there could have been a possibility for new artifacts to be introduced into the data.

The overall process is significantly faster. Re-slicing is a time consuming task. Instead of two re-slicing operations, now only one is required. This is of great importance in real-time fMRI analysis as discussed in the following sections.

From the previous operations, we get normalized and realigned fMRI images. A Gaussian smoothing kernel is applied over these images to get smoothed and normalized realigned fMRI images (whose files are named with the prefix swr).

### Pre-processing for online subject-dependent classification

In this real-time fMRI classification scenario, scan-by-scan processing is performed as opposed to bulk session processing that is seen in offline classification. For real-time applications, the challenge lies in completing the entire pre-processing and the classification before the arrival of the next fMRI image of the brain (for the next repetition time, TR).

**Figure 2:**
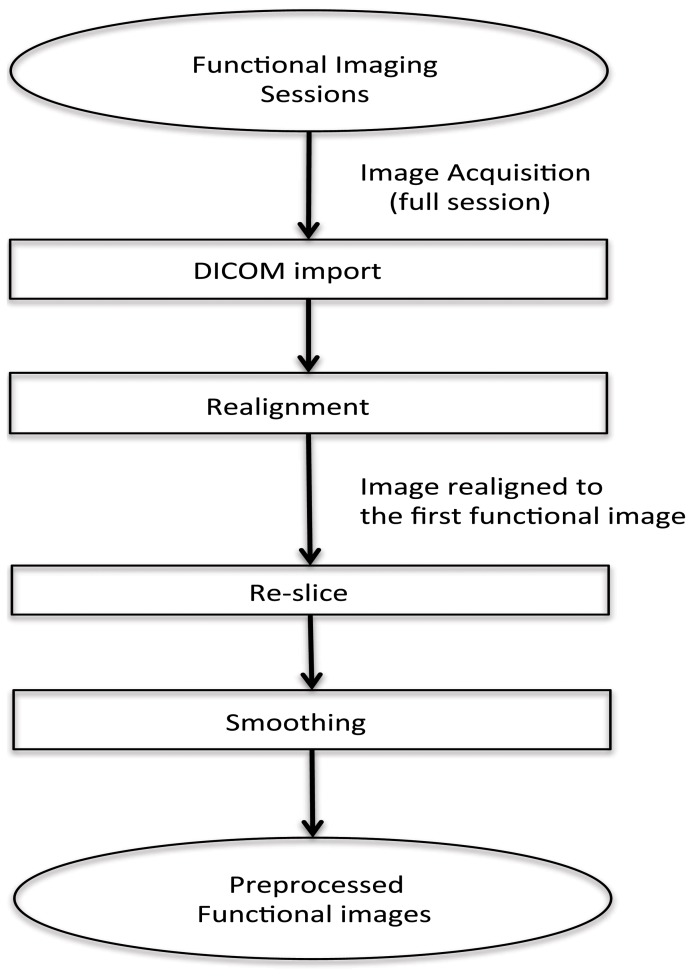
**Flowchart for pre-processing the functional images for online subject-dependent classifier**. (1) DICOM images are converted to the ANALYZE format. (2) Transformation matrix for realignment is then calculated. (3) Re-slicing is performed using the matrix calculated in the previous step. (4) Smoothing of the data is performed.

The method adopted for preprocessing in this case is realignment and smoothing and the scans are processed one-by-one. The process has been explained in the above flowchart (see Figure 2).

#### Pre-processing for online subject-independent classification

To our knowledge, MANAS is the first toolbox that supports real-time (online) SIC. Unlike the online subject-dependent scenario, this involves much more complex processing and a completely different approach.

**Figure 3:**
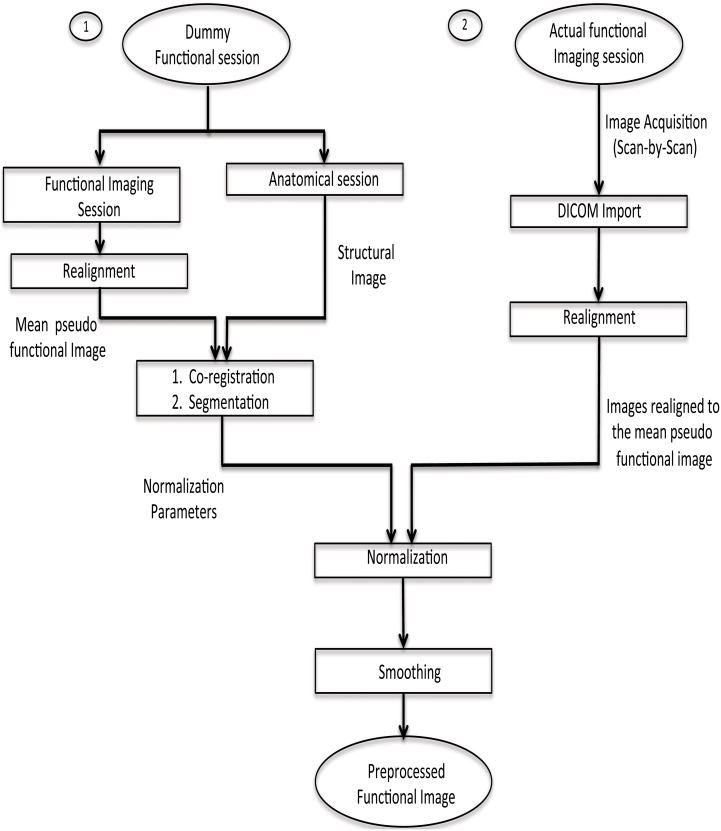
**Pre-processing steps for online subject-independent classification**. (1) Structural scan and dummy functional scan are acquired. Dummy functional provides us with the mean functional image for co-registration. (2) After the co-registration of mean functional image and structural image, segmentation of the structural image is done to calculate normalization parameters. (3) Functional data, pertaining to the task, is converted into analyze format, followed by realignment, normalization using the parameters previously calculated, and finally smoothing is performed.

fMRI scans are to be normalized to a standard space (MNI) using a template of a structural image corresponding to the MNI space. This implies that there should be one structural session before the actual functional session. Co-registration with the structural image is a very time-consuming step, which is not feasible to be performed in real-time. Hence, a dummy functional session is carried out before the actual functional imaging session. The normalization parameters are found from the co-registration of the structural image with the mean functional image of the dummy functional session. Next, when the actual functional session is carried out, its fMRI scans are realigned with respect to the mean functional image of the pseudo functional imaging session. As a result, each scan acquired during the functional imaging session gets normalized with the standard template. This has been explained pictorially in Figure 3. Finally, the normalized realigned functional images are smoothed using a Gaussian FWHM kernel.

#### Feature selection and SVM training

Once the fMRI images have been processed to remove the artifacts and the preprocessing steps are completed, features are extracted and used either for training the classifier or for classifying new images. The feature selection sub-system prepares the fMRI scans for classifier training by extracting the features from them.

**Figure 4:**
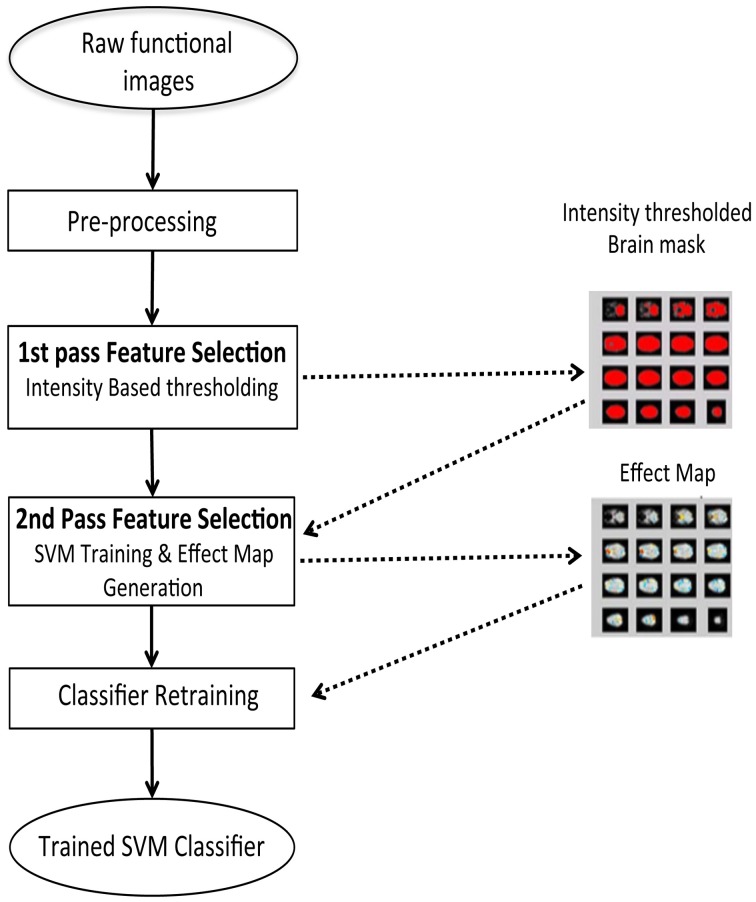
**Two step feature selection mechanism for the online subject-dependent analysis**. Step 1: Intensity based thresholding which removes voxels which do not belong to brain, and Step 2: SVM training and effect map generation which selects the voxels involved in the tasks performed by the subject.

Feature selection is a procedure where informative features from the brain signals are extracted, as an input to the classifier to improve the classifier performance. We performed feature selection in two steps as shown in Figure 4:

1. A first-pass intensity thresholding, and
2. A second-pass voxel selection using effect mapping (Lee et al., 2010).

For the first-pass intensity thresholding, an image of the brain, called intensity threshold mask, is created by removing voxels below a certain BOLD intensity value in a mean image of all the brain scans. An interactive UI allows the experimenter to choose the intensity threshold using a track bar.

For the second-pass feature selection, the training data comprises of only those voxels that pass the intensity threshold mask. However, to correct for the variability of the BOLD signal and to convert it to a signal of zero mean and unit variance, Z-normalization was performed across all the time-course signals at each voxel.

The normalized BOLD values of intensity threshold voxels were used to create input vectors for training SVMs and subsequently to generate an effect Map.

An effect map is a map of effect values (EVs) (Lee et al., 2010) which estimates the effect of the BOLD activation at each voxel on the classifier output by first computing the mutual information (MI) between the voxel and the output, and then multiplying it with the weight value of the voxel as estimated by SVM. MI is derived from relative entropy or the Kullback–Leibler divergence, which is defined as the amount of information that one random variable contains about another random variable (Cover and Thomas, 1991). Hence, the EV *E*_*k*_ of a voxel *k* is defined as:

$$E_{k}=W_{k}I\;\left(X_{k};\;y\right),\;k=1,\;\ldots ,\;M$$

Where *I*(*x*_*k*_; *y*) is the MI between the voxel and the output, *y* is the SVM output after excluding the sign function, *w*_*k*_ and *x*_*k*_ are weight value and activation at voxel *k*, respectively. To reduce the variability of EVs dependence on the BOLD signal, normalized values are used for generating the effect maps. Normalization of *E*_*k*_ was applied as follows:

$$IE_{k}=\text{sgn}\left(E_{k}\right)\text{log}(1+\text{std}(\left|E\right|)),\;k=1,\;\ldots ,\;M$$

where sgn(.) is a signum function, and std(|*E*|) is standard deviation across all the brain voxels.

A threshold can be applied to an effect map at a suitable level based on visual inspection by the experimenter and the resulting map can be used as a brain mask. Finally, an SVM classifier model is then retrained based on the features extracted using this brain mask. This classifier model and the brain mask are stored and later used for classification of the brain states from new subjects or new sessions of experiments.

In the case of real-time subject-dependentclassification, the training data can be collected from the subject before performing the classification session. Such a real-time subject-dependentclassifier was demonstrated in (Sitaram et al., 2011). However, in the case of SIC, the classifier model should be trained on a sufficiently large database of at least 12 subjects' data which can serve as the representative data-set for the whole population. Data from this representative data-set should be first normalized and brought to a standard space before it can be used for training a classifier for SIC.

We implemented this sub-system by building around the C-language implementation of the core engine from SVM-Light (Joachims et al., 1999).

### SVM classification

The brain mask obtained by the two-pass feature selection process is applied to the functional images arriving at each time point (TR), to reduce the data dimension and to choose the most important and informative voxels for classification. This is achieved by SVM to classify the fMRI scans using the weights learnt during the training session.

To classify an individual scan of fMRI data, brain voxels (selected using the brain mask) from each fMRI image can be composed as an input vector *x*^*j*^. SVM determines a scalar class label *Y*^*i*^ (*Y*^*i*^ = sgn (*y*^*i*^ = *w*^*T*^*x*^*i*^ + *b*) = ± 1, *i* = 1, …, *N*, where *N* is the number of input vectors, *T* is the transpose of a vector, b is a constant value, sgn(.) is a signum function).

When the input vectors *x*^*i*^ and the designed labels *Y*^*i*^_*L*_ are taken from the training data set, the weight vector w of SVM is obtained by minimizing objective function *L* of Equation 5 with constraints Equations 6 and 7,

$$L=\frac{1}{2}w^{t}w+C\;\sum_{i=0}^{n}\xi^{i},\text{ with }Y_{L}^{i}\;\left(W^{T}X^{i}+b\right)\geq 1-\xi^{i},\text{ and }\xi^{i}\geq 0$$

Slack variable ξ is introduced to describe a non-separable case, i.e., data that cannot be separated without classification error, and *C* denotes the weighting on the slack variable, i.e., the extent to which misclassification is allowed. A value of *C* = 1 was used in our implementation because of the following reasons. In general, model selection to determine the *C* value is hard to perform in the context of real-time classification due to limitations of time available for SVM training. What is important is that real-time, online classification should work robustly in the majority of participants and sessions. In many previous fMRI classification studies (LaConte et al., 2005, 2007; Mourao-Miranda et al., 2006; Haynes et al., 2007; Soon et al., 2008), *C* = 1 was successfully used. Furthermore, (LaConte et al., 2005) showed that prediction accuracy does not vary a lot with the selection of *C*.

Ideally, SVM output *Y*^*i*^ should be centered about zero, so that when the output is greater than zero the classification is assigned to one brain state, and when it is less than zero it is assigned to the other state. However, due to participants' head movements and other systemic changes, a gradual drift in the classifier output can be expected (LaConte et al., 2007). To remove this bias during online classification in a block design experiment, we subtracted the mean of the SVM outputs during the rest condition block from each SVM output during the active condition block.

The classifier output after the correction of the classifier drift may be used to provide a feedback signal to the subject. A visual feedback such as a graphical thermometer will be used to indicate the correctness of the classification in terms of the bar changing in positive and negative direction. Any other form of visual feedback can also be used by the experimenter. Such a feedback mechanism is the main requirement of neurorehabilitation program.

**Figure 5:**
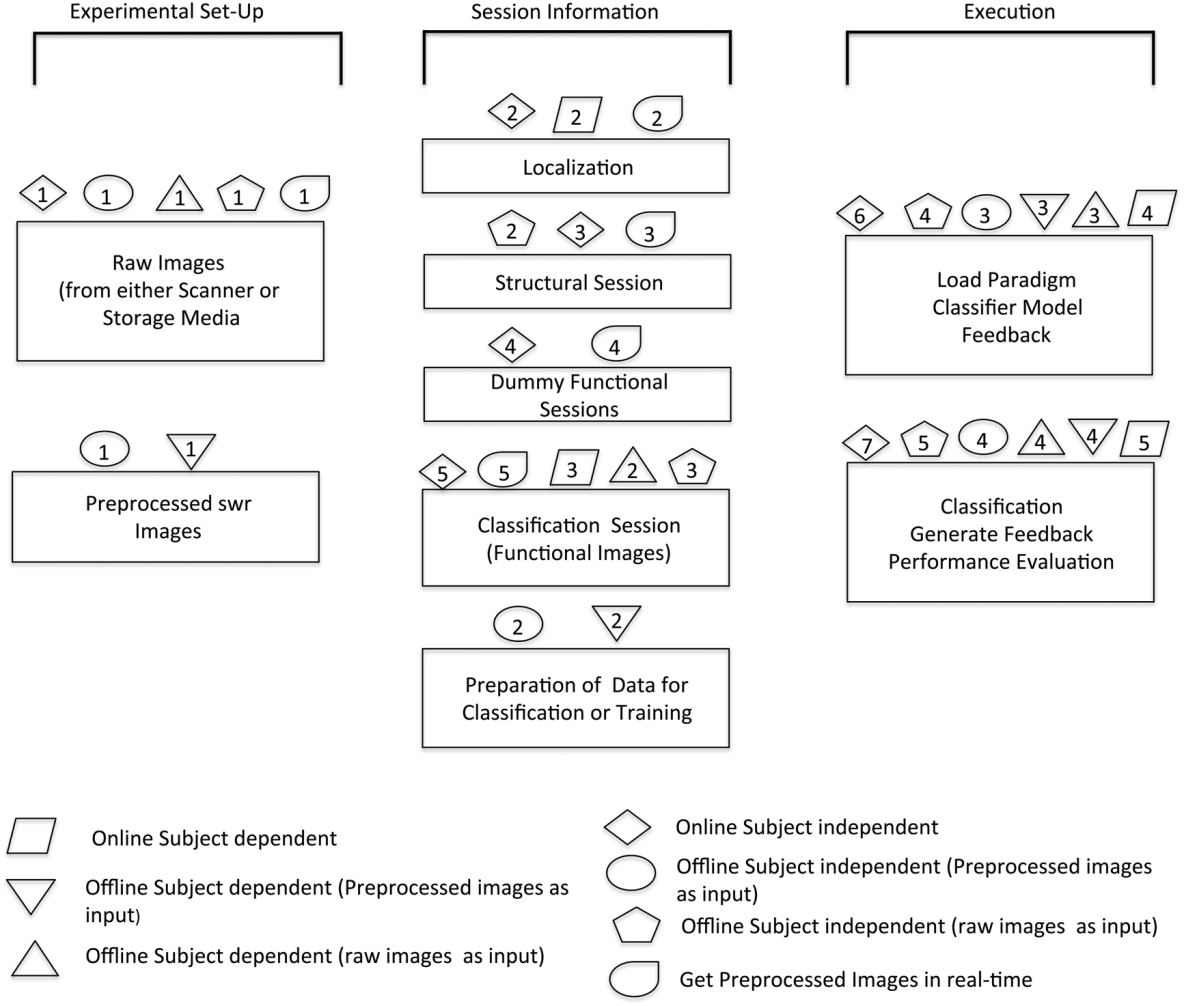
**Overview of software architecture for various modules**. The schematic shows the possible options of analysis that can be done with the help of this toolbox. The sequence in which different steps are performed in order to execute specific modules is illustrated by corresponding symbols.

Modularity is the key feature that has been kept in mind while developing the toolbox. This is important to provide options to the researchers to experiment according to their own requirements. The design of the toolbox is inherently modular. The presented toolbox leverages this power of modular design to provide not only real-time (online) subject-independent analysis, but also other permutations and combinations of online, offline, subject-independent and dependent analysis. However, the process flow in each of these is not the same. Figure 5 shows the software architecture and the various modules used.

## Graphical user interface (GUI)

### GUI for the offline classifier

**Figure 6:**
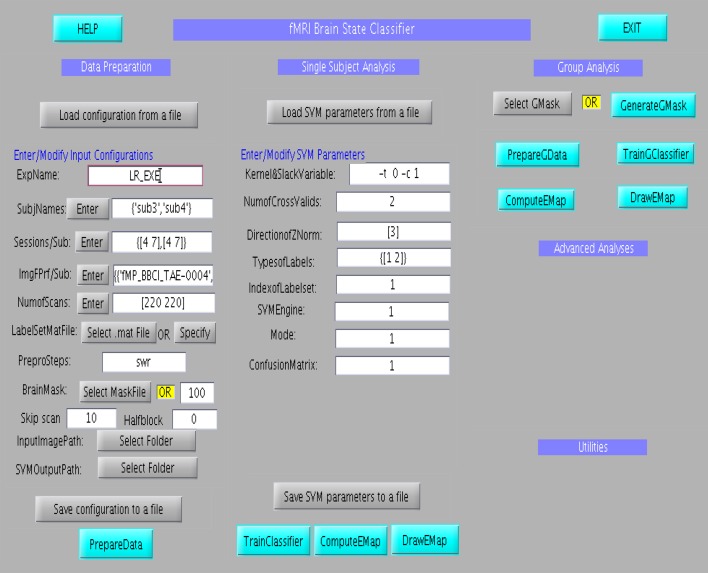
**Graphical User Interface for the offline subject-dependent classification and analysis**. It consists of three main columns namely “Data Preparation,” “Single Subject Analysis,” and “Group Analysis”.

The GUI consists of three main columns namely “Data Preparation,” “Single subject analysis,” and “Group analysis” as shown in Figure 6. The flow of analysis starts with the preparation of data, followed by the setting of parameters of the classifier, and finally ending the classification process, either at the single subject or at group level analysis. In the data preparation step, feature vector, and labelset are prepared according to the experimental paradigm. Information regarding the experiment, including, participant name, number of scans of the protocol, initial file name of the scans, the brain mask to be applied to the data and the paradigm which includes the protocol timings are entered at different fields in the column. Once this is prepared, the parameters of the classifier, such as, kernel and slack variables, number of cross validations and classifier labels is entered in the fields under the Single Subject Analysis column. Once all the data are provided, the classifier can be trained. Effect maps pertaining to the classification will be computed and displayed after the classifier training is completed. Similar to single subject analysis, group analysis is performed by preparing group data using “PrepareGData” button.

### GUI for the online classifier

**Figure 7:**
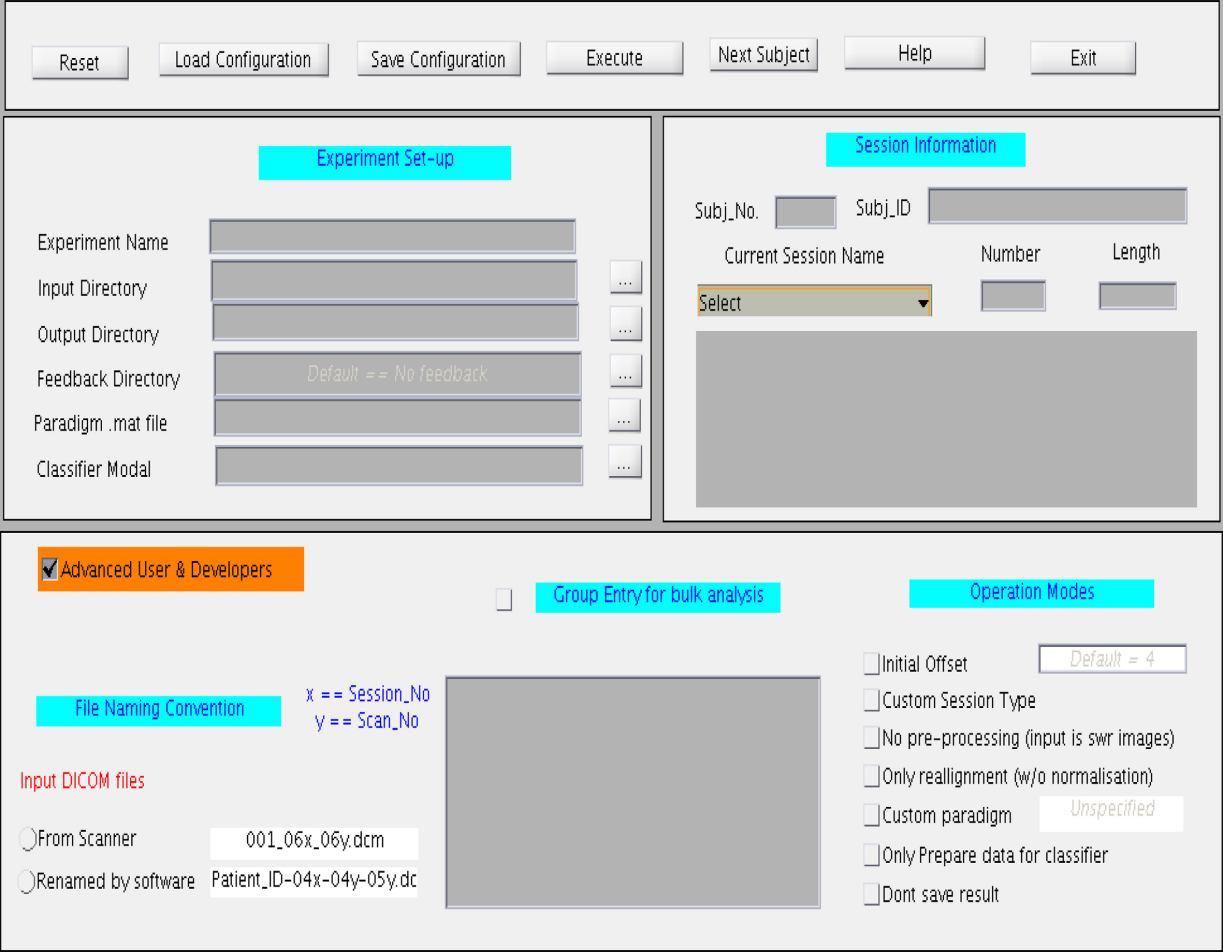
**Graphical User Interface (GUI) for the online subject-independent and subject-dependent classification**. The GUI is divided into three different sections namely “Experimental setup,” “Session information,” and “Advance User and Developers”.

The GUI, shown in Figure 7, is divided into four main sections. The top most section is a menu bar that contains buttons to perform basic functions, such as, reset, save, and load the configuration files, and exit the toolbox. General experiment settings, such as, input and output directories, providing the paradigm file, loading the classifier model, loading the feedback directory, are entered under the column “Experiment set-up”. The column “Session Information” captures subject and session specific information such as subject ID, subject number, session type, session number, and session length. The session length information should be in accordance with the paradigm specified earlier. If the files are read from a storage media, the Subject Name should be same as the Patient_ID prefixed in the file name. This toolbox provides for four types of sessions under the drop-down menu “Session Name”: Functional Localization, Structural Session, Dummy Functional Session, and Classification Session. A menu space is allocated for advanced users and developers, who are interested in customizing their own study, including, classification with or without normalization, providing preprocessed fMRI signals for classification, reading input files from a storage media instead of the scanner in real-time, and so forth.

#### Visualization tools

**Figure 8:**
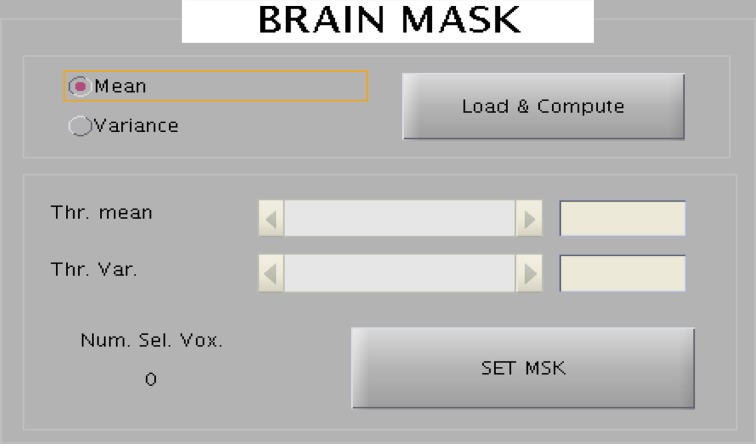
**The wizard shown above is used to create brain mask to reduce the dimensionality of the data**. Two kinds of mask can be created: one represent all voxels in the brain and the other mask corresponds to the voxels belonging to the eyes.

**Figure 9:**
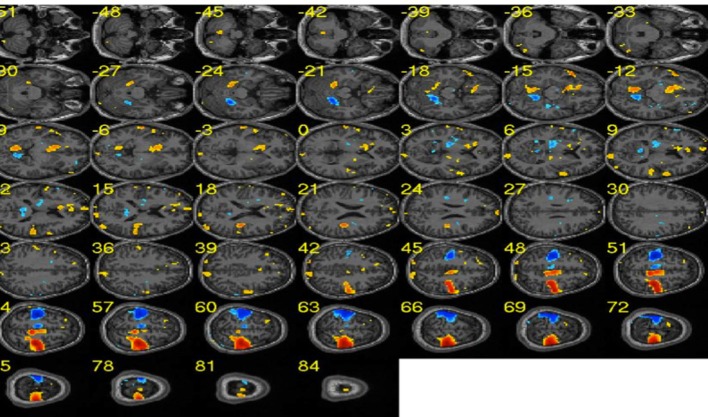
**Sample effect map for a participant performing motor imagery of left and right hands**. Blue clusters depict brain areas related to the right hand execution. Similarly, red clusters show brain areas activated during the left hand execution. [Adapted from Lee et al. (2010)].

The toolbox provides an interactive selection of threshold for generating intensity based and EV based brain masks. The intensity or EV for each voxel for all the slices corresponding to a scan are color-coded and displayed onto a new window. A track bar is provided to select the threshold by moving the pointer. When the experimenter is satisfied with the threshold value, he can save it as a mask for future use. Saved mask can be loaded later through the “Load” button. The track bar and the effect map are shown here in Figures 8, 9.

### Demonstration of the application of the toolbox

Below are three studies, conducted in our lab that is expected to demonstrate the various applications of this toolbox.

### Offline classification of brain states of movement

This was the first experiment to apply the MANAS toolbox to demonstrate the classification of brain states corresponding left or right hand movements. The average classification accuracy for a group of nine healthy subjects for left and right hand movements was over 90% (95.8 ± 1.2%). Our results showed that it is possible to classify brain states with high accuracy with this toolbox. Figure 9 shows the effect map of the left and right hand movements, adopted from our previous publication of these results (Lee et al., 2010).

### Real-time subject-dependent classification of emotional brain states

**Figure 10:**
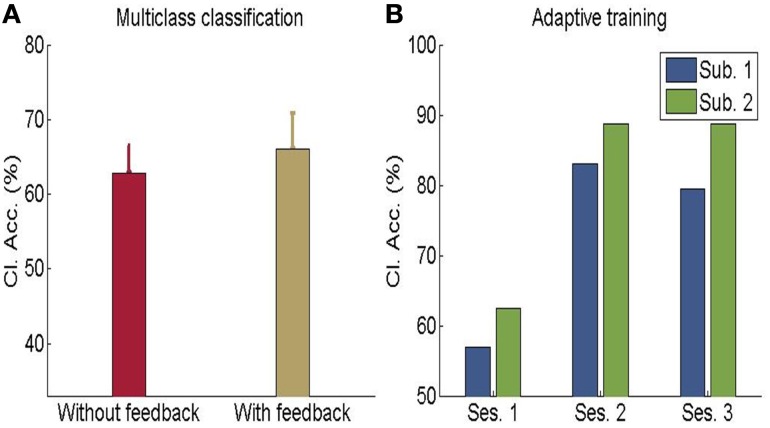
**(A)** Classification accuracy for multi-class emotion data (happy, sad, disgust) with feedback or without feedback (The chance level is 33%) **(B)** Performance of adaptive classifier in participants demonstrating increase in classification accuracy when the classifier was adaptively re-trained by combining previous training dataset with data from every new feedback training run. [Adapted from Sitaram et al. (2011)].

**Figure 11:**
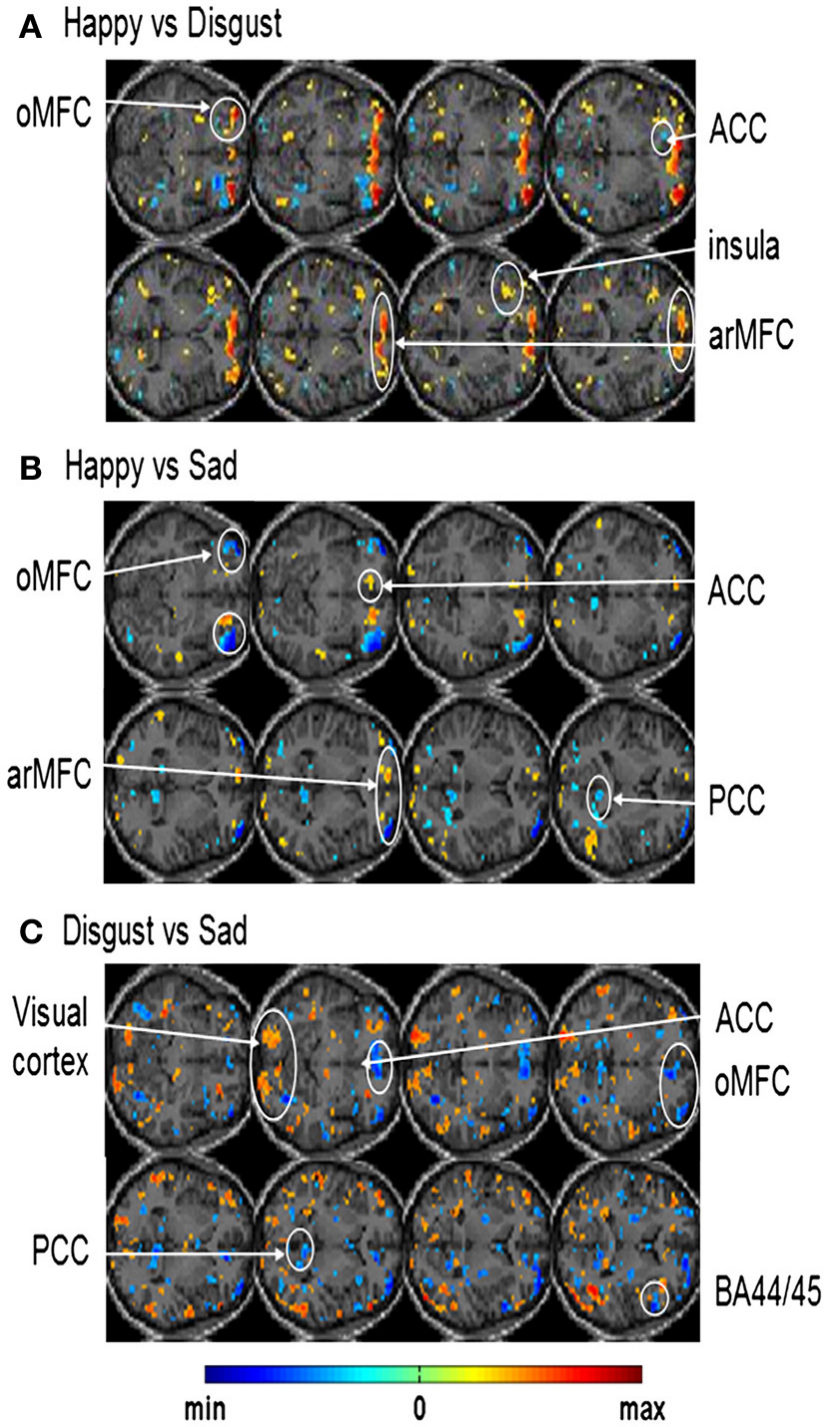
**Effect maps generated from single-subject whole brain SVM classification showing discriminating voxels for: (A)** happy vs. disgust classification **(B)** happy vs. sad classification and **(C)** disgust vs. sad classification [Reproduced with permission from Neuroimage -(Sitaram et al., 2011)].

In another study done in our lab, Sitaram et al. (2011) used the MANAS toolbox to show that an online SVM can recognize two discrete emotional states, such as, happiness and disgust from fMRI signals in healthy individuals instructed to recall emotionally salient episodes from their lives (Figures 10A,B). We reported the first application of real-time head motion correction, spatial smoothing, and feature selection based on a new method called effect mapping. The classifier also showed robust prediction rates in decoding three discrete emotional states (happiness, disgust, and sadness) in an extended group of participants. Multivariate brain mapping performed using the effect mapping method, available as part of the toolbox, shows greater involvement of the prefrontal cortex in the emotions (Figure 11).

### Real-time subject-independent classification of emotional brain states

**Figure 12:**
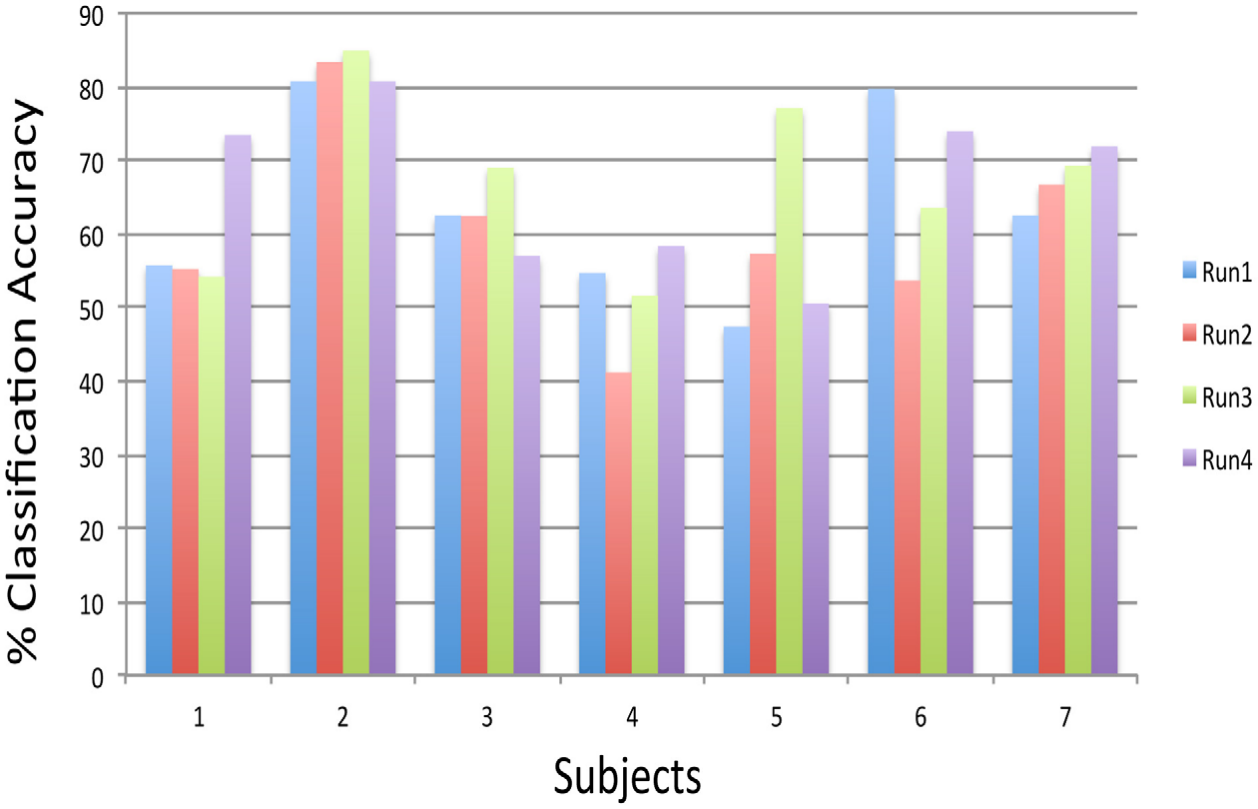
**Subject-independent Classifier (SIC): classification accuracies of subjects performing happy and disgust imagery guided by the feedback calculated from a model, which was built on 12 subjects data, across four feedback runs**. The chance level is 50% (Rana et al., 2012).

Preliminary experiments on healthy human volunteers in our lab (Rana et al., 2012) have indicated the possibility that a subject-independent classifier, built on data from a group of volunteers, can be used for real-time classification and feedback of emotions of a new participant, performing alternating blocks of happy and disgust mental imagery (Figure 12). Multiple runs of feedback training of a volunteer on such a classifier showed increasing classification accuracy, indicating that the subject gradually learned to produce brain states similar to those of the healthy group. These pilot data suggest that such a system might help patients to correct maladaptive brain states by neuro-feedback training. Ongoing studies in our lab would need to confirm these findings.

## Discussion

### Software features

#### What can the toolbox do?

The toolbox has been able to meet all the design objectives and has provided a simple interface for classification of brain states using fMRI signals. Incorporating the concept of modularity in the software design has been the key factor that has expanded the ambit of its use and made it flexible for application in a variety of scenarios.

Given that the toolbox is mainly targeted for use by researchers and experimenters, it is expected that they will apply this tool for different experiment by creating new classifier models and test its performance in decoding brain states. For this, we have provided an input field through which the user of this toolbox can choose a new model. Moreover, the toolbox can be used for both two-class classification as well as multi-class classification, for example as illustrated in Sitaram et al. (2011) for real-time decoding of emotional imagery.

The toolbox provides for many customization options. A user who is just interested in checking the classifier accuracy without simulating over the whole experiment can straight away provide the pre-processed (smoothed, normalized, realigned) images as input to the classifier. The experimental paradigm can be altered according to the requirement of the experiment, which opens the scope of the toolbox for various applications such as decoding motor imagery, deciphering emotional states of the brain, neuro-rehabilitation, lie detection, and so forth.

The toolbox provides an interactive user interface for selecting directories, visualizing (Intensity maps, effect maps, and results), saving the data and results. The configuration, i.e., the values of the fields in the GUI can also be saved, and later loaded, with a simple “Load” button. A track bar is provided to easily set the threshold in Intensity maps and effect maps. The feedback, besides being shown to the subject, is also visible to the experimenter for reference.

The time taken per functional scan for completing the full process starting from image acquisition, preprocessing and classification is around 1 s for a typical personal computer (the actual time varies depending on the hardware limitations and the session size). It shows that real-time preprocessing (including normalization) and classification is indeed realizable under usual conditions.

### Future directions or the limitations of the toolbox

However, many functionalities and features are open for future improvement. For example, in the current toolbox, a “paradigm.m” file is required to be provided for specifying the paradigm of the experiment being conducted. A user interface can instead make the task much easier and faster. The results and the messages are currently being displayed on the MATLAB command. A display panel on the main GUI will be much more convenient to the user.

The major application of the toolbox is to use SIC in neuro-rehabilitation. In order to implementing it, we have to normalize the functional images, but in case of neurological patients with lesions, at this point in time, it is challenging to perform this normalization in an automatic mode. Instead, we propose that normalization be performed offline with another tool or expertise meant for this purpose, and only import the Affine Transformation Matrix into our toolbox toward performing online SIC.

Currently this toolbox has been developed based on the SPM2 software that reads files only in ANALYZE format (^*^.hdr and ^*^.img). Support for other file formats like NIFTI will be done in the future releases of the toolbox. Currently, this toolbox requires MATLAB version 6.5 SPM2 and is not supported on the higher versions of MATLAB. However, as classifier training is done using the SVM-Light library (Joachims et al., 1999), the training module needs to be run on MATLAB version 7.1 or higher. Because of these compatibility issues, the classifier cannot be trained using the GUI alone. It only prepares the data for the training and the actual training happens on a separate MATLAB instance having version 7.1 or above. In the future, by making this toolbox compatible with SPM8, we would be integrating the training module with the rest of the GUI so that the classifier can be trained by a single click.

Due to the limited resources, the authors would not be able to distribute this toolbox in a webpage. However, interested users can acquire the toolbox by sending a request to the authors.

### Conflict of interest statement

The authors declare that the research was conducted in the absence of any commercial or financial relationships that could be construed as a potential conflict of interest.
